# Context-dependent memory recall in HMD-based immersive virtual environments

**DOI:** 10.1371/journal.pone.0289079

**Published:** 2023-08-04

**Authors:** Mária Chocholáčková, Vojtěch Juřík, Alexandra Ružičková, Lenka Jurkovičová, Pavel Ugwitz, Martin Jelínek

**Affiliations:** 1 Department of Psychology, Faculty of Arts, Masaryk University, Brno, Czech Republic; 2 Behavioral and Social Neuroscience Research Group, CEITEC – Central European Institute of Technology, Masaryk University, Brno, Czech Republic; 3 Department of Neurology, St. Anne’s University Hospital and Medical Faculty of Masaryk University, Brno, Czech Republic; 4 Department of Geography, Faculty of Science, Masaryk University, Brno, Czech Republic; 5 Institute of Psychology, Czech Academy of Sciences, Brno, Czech Republic; Politecnico di Torino, ITALY

## Abstract

The article introduces an original VR-based experiment which explores context-dependent memory recall in humans. It specifically examines the recall of correct and falsely induced semantic memories. With the aid of VR head-mounted displays, 92 students of psychology were placed in a computer-generated indoor virtual environment and asked to memorize the presented lists of words. Afterwards, the participants were placed in the same indoor virtual environment or an alternative outdoor virtual environment and asked to recall the words. The number of correct and falsely induced words was then measured. On average, women recalled significantly more correct words from the list than men, regardless of the environmental context. Despite the assumptions, we did not observe a separate effect of exposure to different environments during learning and recall of material on memory performance. Likewise, we did not detect any effects of the learning context or biological sex in the case of the production of false memories. These results provide a novel insight into previous knowledge regarding the memory processes that occur in virtual environments. Although we failed to confirm the role of context in recalling learned material in general, we found a hint that this context might interact with specific memory processes of biological sexes. However, the design of this study only captured the effect of changing the environment during memory recall and did not address the role of specific context in remembering learning material. Further research is therefore needed to better investigate these phenomena and examine the role of biological sex in context-dependent memory processes.

## 1. Introduction

False memories can be considered incorrect or completely fabricated content recollected from memory, which can be vivid, detailed and held with high confidence [1]. Most studies have focused on manifestations of false memories in episodic memory. The first successful attempt to evoke false memories in semantic memory using only word lists was done by Deese [2] who created a total of 36 lists, each containing twelve semantically-connected words and evoking one strongly associated but not-presented word–a so-called “critical lure”. Each set of words was presented auditorily and was followed by immediate free recall. The sets of words that successfully evoked false memories were later used by Roediger and McDermott [3]. Unlike Deese [2], they focused not only on free recall, but also on word recognition. With reference to these studies, the method of using sets of semantically connected words to create false memories of learning a non-presented critical lure is known as the Deese-Roediger-McDermott paradigm (DRM paradigm; [4]). The creation of false memories in this paradigm has been observed under a variety of conditions [5, 6], where the production of false memories was found to be persistent even in translated lists, and where strongest effect was observed when words were presented in the mother tongue [7, 8]. In their dominant language as opposed to the non-dominant one, people are more likely to create false memories. Although, when speakers are equally fluent in both languages, there is no difference in false memories between two languages [9].

In the context of the DRM paradigm, memory is affected by semantic associations, feature overlap, phonological associations, and orthographic/lexical association, and these effects are sufficiently age-invariant [10]. In previous studies using the DRM paradigm, the learning of words has often been accompanied by a specific piece of contextual information, such as images of visually or conceptually similar objects or audio recording of a female or male voice reading the words [11, 12]. It is generally believed that specific context, and therefore contextual information, affects memory performance [13]. Critical lures are thought to be spontaneously generated during the learning phase, and the reinstatement of the study context may boost monitoring processes and thereby recall accuracy [14]. An alternative theory suggests that there are two traces encoded when studying items–verbatim traces, preserving contextual information about items, and gist traces, encoding the overall theme of the studied category [15]. Critical lures are consistent with the gist trace. Contextual reinstatement may be responsible for strengthening verbatim traces of studied items. Better recollection from memory in the case where the same contextual information from the learning phase is also available in the remembering phase is called context-dependent memory (CDM) [13]. This effect was demonstrated by Godden and Baddley [16] when they had divers learn lists of words underwater and on land, and was further confirmed in several other studies (see [17], for a review). The term “context” covers a wide range of factors ranging from external aspects, such as the room, the music, the smell, or the background colour, to internal factors such as mood and emotional, mental, and physical state [17]. Nevertheless, context is not helpful in recognition tests–studied objects put between unstudied ones are considerably more effective memory aids than contextual information. [18].

Reinstating the word encoding context during the memory test, relative to testing with nonreinstated context, considerably increases participants’ performance [19]. This is also supported by the results of a neuroimaging evidence, showing that neural reinstatement of encoding patterns only improves memory when the retrieval and encoding context match, whereas neural reinstatement is detrimental when these contexts differ [20]. Additionally, environmental scene context reinstatement during recall attempts improves recall performance regardless of whether this encoding context is presented or only imagined [21]. The significant role of context in learning material in virtual reality (VR) was demonstrated by a study focused on highly challenging material, i.e., foreign vocabulary in two phonetically similar languages, which proved that context supports higher retention of foreign words [22]. Although, this effect also depended on other factors such as sense of presence, a separate context for each of the languages and delayed recall. Contrastingly, according to another study, it does not seem that the variability of the VR contexts in which participants learn the material has an effect on improving nor impairing memory recall [23]. It is not completely clear which of the contextual factors have the greatest impact on a memory performance, because the results are not consistent and there is a great variability between studies (see [17]). However, there is a general belief that visual contexts with meaningful content, rich in informational stimuli, are more likely to be encoded into memory alongside the content [17, 24]. This kind of context includes visual material such as photos or videos [25].

VR technologies allow us to study cognitive phenomena in well-controlled realistic conditions [26–28], since they promote the activation of cognitive mechanisms similar to those that occur in the real world [29–32]. Contemporary virtual interfaces give users a strong feeling of presence, as well as fast and accurate sensory feedback at various levels of interactivity [33]. Higher immersivity is intended to enhance abstract mental activities such as learning [34, 35]. The examination of differences in the use of memory palaces employing VR head mounted display (HMD) compared to traditional desktop display use brought the finding that recall accuracy was better in the HMD condition, possibly due to the superior sense of spatial awareness [36].

With regard to the use of head-mounted displays (HMDs), Wälti, Woolley and Wenderoth [37] reproduced several semantic CDM studies in which they focused on the contextual dependence of memory on visual context. They used different colours, photos of natural scenery, VR environments or flickering as the background for the presented words. However, in this case, a very small effect on improving memory performance was found for local flickering cues only. In contrast to the Wälti, Woolley and Wenderoth who used backgrounds that did not allow interaction in VR, Shin and colleagues [38] investigated how interaction with the VR environment contributes to context-dependent memory in addition to the reinstatement effect. Their results suggest that context that activates existing schema enables improvement of item recall only if the items have previously been integrated into the given context (e.g., by judging them to be "useful" in that context). Moreover, few studies have specifically examined the influence of context on the induction of false memories within the DRM paradigm [39, 40]. These studies have suggested that there is an effect of context reinstatement not only on correct memory recall performance but also on false memory reduction. Specifically, reinstating the study context at the test increased memory accuracy in correct word recall as well as reduced the induction of false memories. However, as we have demonstrated above, the research provides inconsistent evidence regarding the principles of the context-dependent memory. This subject requires further examination, particularly utilizing visually salient and highly realistic virtual environments. Besides the traditional view suggesting an important role of an active post-encoding consolidation process in fixing memories into long-term storage, we use a more current alternative approach, the contextual binding (CB) theory, assuming that episodic memory reflects the capacity to recall the context in which objects or items were previously encountered [41]. Since very little research combining VR and context-dependent false memory induction (including false memories) has been done so far, we aimed to bring more insight into this issue in the present study.

In order to study semantic memory recall and false memory induction in VR, we adopted the methodology by Roediger and McDermott [3] and adapted it for the VR-based original experimental scenario. The research aimed to answer two basic research questions: first, whether the correct recall of the material is influenced by the study context; and second, whether exposure to a different context during recall than during learning induces more false memories compared to re-exposure to the same context. Referring to the previous research we discussed above (e.g., [19, 21, 39, 40]), in this study we expected that the number of correctly recalled words would significantly increase when participants recalled them in the virtual environment they had learned them in, and that the false memories induction would be significantly enhanced by the alternative virtual context, when compared to the original learning context. Additionally, we hypothesized that CDM performance between females and males may differ depending on task demands. Previous studies on memory have emphasized sex variability between memory types such that males tend to have better spatial memory, whereas females tend to have better memory performance in terms of autobiographical and semantic memory [42, 43]. Human sex hormones–estrogens and androgens–may have different effects on specific forms of spatial and verbal memory, depending on factors like age, sex, or task demands [44]. Nevertheless, research rarely focuses on differences between biological sexes in memory performance. In light of this, we also aimed to examine and quantify the performance difference between the two biological sexes.

## 2. Methods

### 2.1 Participants

Data were collected from 92 healthy participants, mainly students of psychology and humanities at Masaryk University (33 males, 59 females, med = 22 years; SD = 4.61). All participants were proficient in the Czech language (speaking and reading), however, for some it was not the mother tongue, as we included two nationalities: Czechs (N = 74) and Slovaks (N = 18). A prerequisite was that participants had no serious health or visual disorders or any other medical impediments that would disqualify them from the study. Wearing glasses was not deemed to be an impediment and participants were instructed to use contact lenses where possible. Participation in the experiment was voluntary. Participants did not receive any compensation for their participation. Prior to the research, informed consent was presented and the participants were informed that the research was designed to examine memory recall performance under specific environmental circumstances, but the specific purpose of the study was not explicitly explained until the end of the experimental session. Participants were made aware of potential feelings of motion sickness when using HMDs and the fact that they were allowed to withdraw from the experiment session at any time without providing any justification. Then, they were randomly assigned to an experimental group or a control group. Given the relatively static nature of the virtual environments presented, no participants reported any serious sickness caused by virtual reality exposure (usually referred as cybersickness [45]). None of the participants withdrew from the experiment during the testing and none of the recorded data was excluded. The study was approved by the Ethical panel of the Department of Psychology of the Faculty of Arts of Masaryk University. The study was conducted in accordance with relevant guidelines and regulations following the principles of the Declaration of Helsinki.

### 2.2 Material and stimuli

On the basis of previous studies [3], for the purposes of this study we selected five word lists (see Table 1) which are commonly used in the DRM paradigm [46] and are most likely to induce in participants a false memory of a critical lure. The lists were translated from English into Czech and were modified to fit better in the context of the Czech language (see Table 2). Some words were removed, because they are not ordinarily used in Czech or because there was no connection between the word and the critical lure, and some words were replaced with more commonly used Czech equivalents. Each of the five lists contained eleven words intended to evoke exactly one critical lure, i.e., (1) window, (2) sleep, (3) doctor, (4) sweet and (5) chair. All five word lists were presented as one, but with five second pauses between the individual sub-lists.

**Table 1:** Original word lists taken from Stadler, Roediger, McDermott [46].

window	sleep	doctor	sweet	chair
door	bed	nurse	sour	table
glass	rest	sick	candy	sit
pane	awake	lawyer	sugar	legs
shade	tired	medicine	bitter	seat
ledge	dream	health	good	couch
sill	wake	hospital	taste	desk
house	snooze	dentist	tooth	recliner
open	blanket	physician	nice	sofa
curtain	doze	ill	honey	wood
frame	slumber	patient	soda	cushion
view	snore	office	chocolate	swivel
breeze	nap	stethoscope	heart	stool
sash	peace	surgeon	cake	sitting
screen	yawn	clinic	tart	rocking
shutter	drowsy	cure	pie	bench

**Table 2:** Translated and modified lists of words for Czech language conditions.

okno (window)	spánek (sleep)	doktor/lékař (doctor)	sladký (sweet)	židle (chair)
žaluzie (sunblind)	šlofík (nap)	pediatr (pediatrician)	med (honey)	stůl (desk)
balkon (balcony)	dřímota (slumber)	sestřička (nurse)	hořký (bitter)	křeslo (armchair)
parapet (parapet)	postel (bed)	stetoskop (stethoscope)	cukr (sugar)	sedadlo (seat)
rolety (roller blinds)	probudit se (to wake up)	chirurg (surgeon)	cukřík (candy)	stolec (throne)
závěsy (hinges)	ospalost (drowsiness)	pacient (patient)	kyselý (sour)	stolek (table)
dveře (door)	únava (fatigue)	poliklinika (polyclinic)	bonbón (sweetmeat)	gauč (sofa)
sklo (glass)	odpočinek (rest)	zubař (dentist)	zákusky (desserts)	sedět (sit)
plast (plastic)	chrápaní (snoring)	lék (medicine)	čokoláda (chocolate)	pohovka (settee)
vitráž (stained glass window)	bdít (keep awake)	zdraví (health)	chuť (taste)	lavička (bench)
záclona (curtain)	sen (dream)	léčba (cure)	dort (cake)	podsedák (booster seat)
výhled (view)	zívání (yawning)	nemocný (ill)	nápoj (drink)	dřevo (wood)

### 2.3 Procedure

On arrival at the laboratory, participants were welcomed and acquainted with the experimental procedure and other aspects of the research, including the possible risks (particularly cybersickness). The participants had received written instructions in advance by e-mail and were informed again just before the experiment. Subsequently, after recapitulating all the information, an informed consent was signed. Afterwards, the HMD VR technology was introduced and participants had the opportunity to try it on. Participants were seated on a chair beside a table to prevent possible nausea. When participants were feeling comfortable wearing the HMD, they were teleported to the virtual environment. The whole experimental run took place in VR and participants did not take the HMD off in any part of the experiment, so that the VR environments were the only ones participants were exposed to during the experiment [47].

In order to stay as close as possible to the original procedure used in the DRM paradigm and to control for potential confounding variables, participants were tested separately and were not allowed to interact with the virtual environments. Participants were able to look around in the virtual exposition by moving their heads, which manifested in changes in the virtual camera position. With regard to previous studies in the field [13, 16, 17], this experiment used free memory recall instead of recognition tasks, as it was assumed that studied items placed between non-studied items in recognition tasks are much stronger memory aids than contextual information [17]. Commonly used words that are easy to remember are presumably more likely to be paired with contextual aids/triggers [48], which we believe promote context-dependent memory recollection.

**Fig 1:**
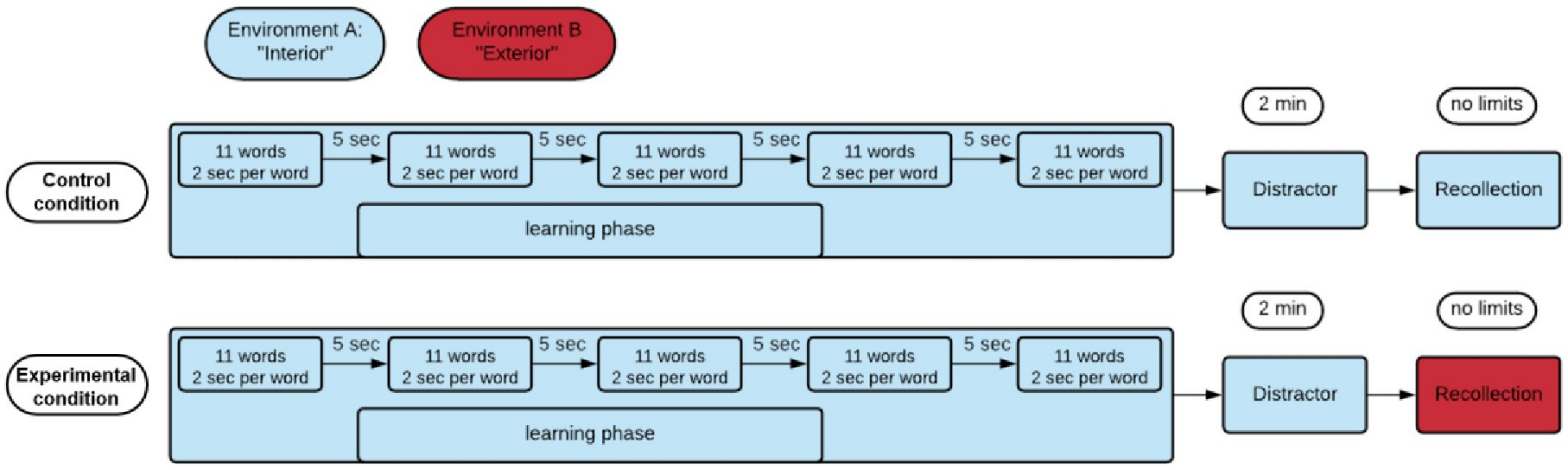
Experimental scheme (control group consisted of 47 participants, experimental group consisted of 45 participants). All participants first learned lists of words in five trials. In each trial, a list of 11 words was presented. Each word appeared for 2 seconds against the background of a virtual indoor environment. There were 5 second breaks between trials. After completing all the trials, the participants solved a mathematical problem, which could last a maximum of 2 minutes, but was often solved in just one minute. In the following recollection phase, the participants had to freely recall as many words as possible from the lists they had learned. Participants in control condition had to recall the words in the same environment in which they learned them, whilst those in experimental condition were exposed to a new outdoor environment.

As the study was a between-subjects experimental design, participants were pseudo-randomly assigned to two conditions, taking into consideration reported biological sex. Both groups were put into the virtual Environment A (see Fig 1), where they were asked to learn a series of visually presented words. Since the experiment examined context-dependent memory recollection and false memory induction, we manipulated the exposition of the specific environmental context for the memory recollection phase.

#### The task

The original design of Roediger and McDermott [3] was adapted and optimized for application in a VR-based study. The experiment consisted of two essential parts: the learning phase and the recollection phase. During the learning phase, participants were asked to look around the room (Environment A) in order to familiarize themselves with the environment. Although sensory stimulation was limited to the visual aspect, they were able to look around them and their entire field of vision was stimulated, which was expected to further promote physical environmental fidelity [49] and the participants’ sense of agency [50], thereby enhancing ecological validity. Respondents were not explicitly instructed to form associations between the environment and the words that would be presented to them later. When respondents felt they had familiarized themselves with the room, they signaled to the experimenter to begin presenting the words, which were displayed via the HMD. The words did not appear in a specific place in the room but followed the person’s gaze in the center of the virtual scene. Each word was presented separately for two seconds. There were five thematic sub-lists, each of which consisted of 11 words (55 words in total). There was a short, five-second pause between sub-lists. All five sub-lists were presented in the same order for all participants. At the end of the learning phase, there was a distractor (participants were asked to report if a visually presented mathematical equation is correct or not), which was again shown via the HMD in Environment A. Its only purpose was to distract the attention of participants and prevent the intentional repetition of the learned words. Its correct solution was not necessarily requested to proceed with the experiment. The maximum time to solve the task was 2 minutes, although it often took the participants about a minute.

After the distraction, participants in the control group stayed in the environment in which they had initially learned the words (Environment A), while the experimental group was placed in an alternative outdoor environment (Environment B). Once introduced to their specific environment, participants were instructed to look around and begin freely recalling the memorized words by saying them out loud. The words could be spoken in any order and could be repeated, but they could not be made-up or guessed. The memory output was recorded on a recording device and thereafter transcribed. During the transcription, the repeated words were removed. Words that participants had verbally expressed some uncertainty about (e.g., by declaring that they were not sure) were not counted to avoid including guesses in the data. We did not set a specific time limit for remembering words, but it usually took between one and three minutes. The recollection session ended as soon as the participant was not able to recall any more words. At the end of the procedure, the HMDs were removed, the participants were thanked for their time and debriefed.

### 2.4 Virtual user interface

The HTC Vive Pro HMD was used for presenting the virtual context. This device’s display hardware consists of two 3.5-inch (8.89 cm) AMOLED screens with a resolution of 1440 x 1600 pixels per eye and a refresh rate of 90 Hz [51]. We also utilized the HMD’s capability for movement tracking by linking the head movement of the participants to the rotation of the virtual camera of the scene observed in VR, so as to enhance the fidelity of the exposition. The VR setup consisted of the HTC Vive Pro VR headset connected to the computer by a cable, keyboard and mouse. Optimization of the hardware and scene ensured a seamless VR experience. A PC equipped with an Intel i7 8700 K CPU and Nvidia GTX1070 GPU rendered the scene at 90 frames per second. The virtual environment was modeled and launched in a Unity graphic engine.

Two meaningful yet significantly different environments with approximately the same number of visual stimuli were adopted from Unity Asset Store [52]. The Unity Asset Store is the official asset repository of the Unity 3D engine. Unity, like most contemporary 3D engines, provides an open 3D graphics pipeline with the ability to adjust, modify and reuse various virtual environments and their components [53]. Thus, we modified the original environments taken from the Asset Store to make them fit the purposes of our study, especially focusing on the composition of environments. In the first environment (Environment A) the participant was seated indoors on a couch with a TV and conference table directly in front of him/her. There was other equipment and furniture in the background around the entire visual field. The second environment (Environment B) was an ancient city courtyard. In this case, there were two lion statues in front of the participant, with a background consisting of the surrounding buildings and some accompanying vegetation.

To ensure the virtual experiment remained accurately operationalized, as per Fig 1, *Toggle Toolkit* [54] was used. This toolkit consists of a series of scripts that allow for adding dynamic elements into 3D visualizations. Even though this experiment did not use many forms of interaction, the toolkit was used to facilitate the basics, i.e., to present the text stimuli in a given order and timing, and to transition from Environment A to Environment B. Since the toolkit produces log outputs of its own, its accuracy throughout the experiment could be verified by checking the actual timings of the events it handled.

### 2.5 Data analysis

The data were analyzed using linear mixed effects modeling (LMER) [55] separately for correctly recalled and falsely induced words. The number of correctly recalled words from the presented list and the number of falsely induced words (critical lures) were treated as dependent variables (responses). Correctly recalled words were scored 1; the false recalls where they met the definition of the critical lure (in our case: window, doctor, sleep, sweet, and chair) were also scored 1. Both total scores were obtained by summing the corresponding category of words (correctly recalled or critical lures) and were modeled separately. The correctly recalled words were distributed asymmetrically, so we applied logarithmic data transformation logit as a link function. For the responses, the experimental condition and sex were considered fixed factors. The original word lists [46] were translated into the Czech language. Since the language proficiency of some respondents might have affected their performance in the tasks [7] and two nationalities (Czechs and Slovaks) took part in the experiment, the native language of the participants was treated as a random intercept within the model. The Czech and Slovak languages are very similar [56] and both nations understand each other perfectly well in terms of semantics. Nevertheless, there may be differences in the connotations and mental representations associated with Czech words. All data were processed using R software [57]. R packages lme4 [58] and lmerTest [59] were used to execute linear mixed effects models. The final models for interpretation were chosen based on the comparison of their relative fit to the data, as determined by AIC and BIC. The *p*-values for fixed effects were obtained using Satterthwaite approximation for degrees of freedom. Reported confidence intervals were obtained via bootstrapping [55], 100000 iterations for LMER.

## 3. Results

### 3.1 Correctly recalled words

**Fig 2:**
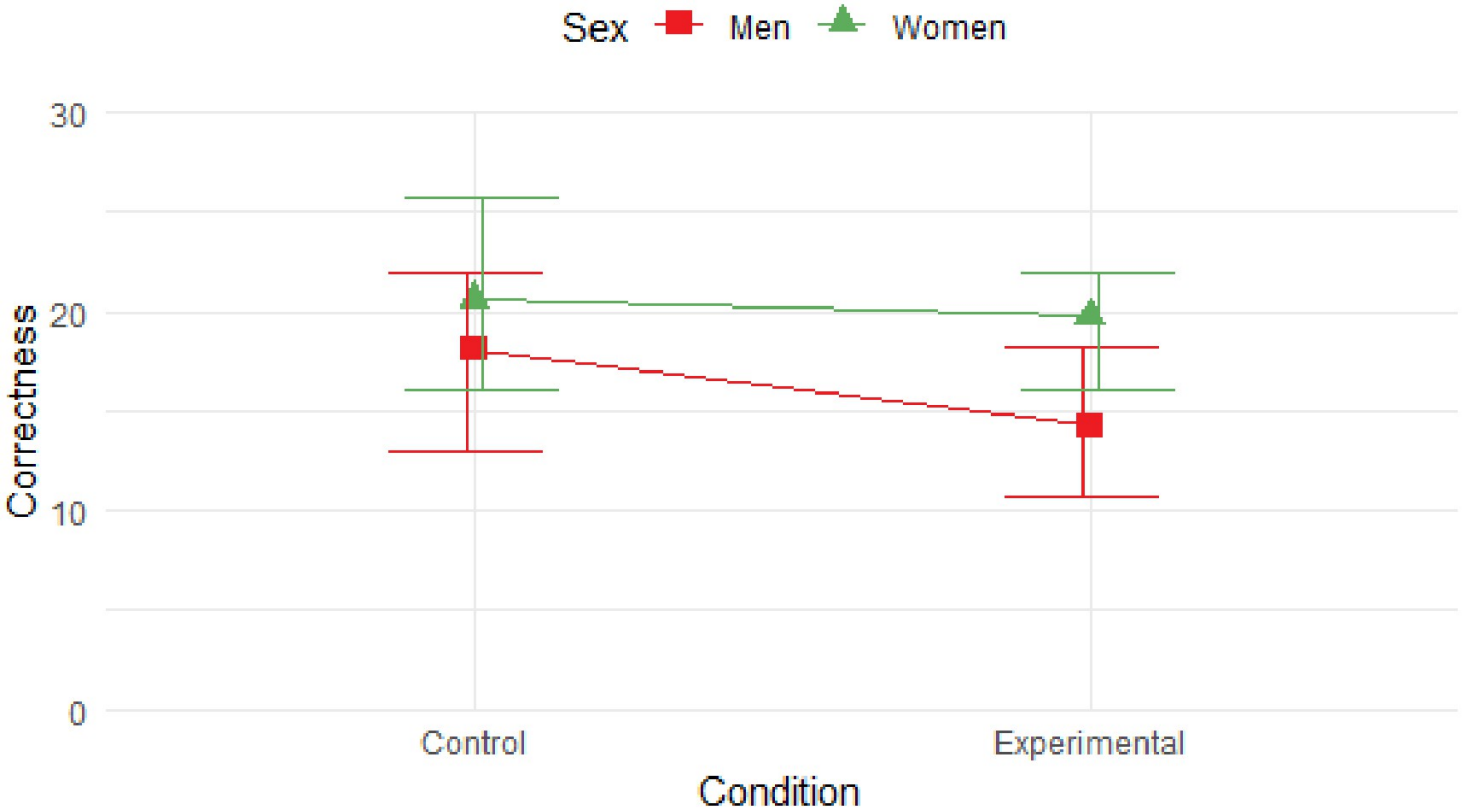
Average correct recall of words according to sex and condition. Compared to men, women remembered a significantly larger number of words in both control and experimental conditions, although the comparison of experimental conditions does not refer to a significant difference in memory recollection. The Y-axis represents the sum of all correctly remembered words by the participant, while the X-axis specifies the score according to the experimental condition. Error bars here represent the range of values that contain the middle 50% of the data. Overall, women recalled significantly more words than men in both conditions.

The linear mixed effects model for correctly recalled words showed significant main effects for sex (*t* = 2.955; z = 0.217; *p* = .004; 95% CI [1.062, 6.262]) but not for the experimental condition (*t* = -1.832; z = -0.128; *p* = .070; 95% CI [-4.649, 0.311]). More specifically, women recalled the words from the list with greater accuracy than men. Men recalled 16 words on average (m = 16.24; SD = 5.77) compared to 20 words on average for women (m = 20.20; SD = 6.28). No other significant main or interaction effects were found. As seen in Fig 2, the recall of participants in the alternative environment was not different in terms of the number of correctly recalled words. This means that participants present in the same environmental context did not tend to recall significantly more words from the original list (N = 47; m = 19.72; SD = 6.49) than participants placed in an alternative virtual scene (N = 45; m = 17.80; SD = 6.156). Frequencies per sex and conditions are summarized in Table 3.

**Table 3:** Summary of central tendency measures in correctly recalled words.

Sex	Condition	N	Mean	Median	SD
Men	Experimental	16	14.250	13.5	4.892
	Control	17	18.118	17	6.040
Women	Experimental	29	19.759	20	5.962
	Control	30	20.633	19.5	6.657

Visual inspection of Fig 2 shows that apart from the overall difference in memory performance between the biological sexes, no other effect of the experimental condition or its interaction with the biological sex of the participants was found.

### 3.2 Critical lures—Falsely recalled words

The linear mixed effects model was used for falsely recalled words identified as critical lures, but no significant effects were demonstrated regarding experimental condition (*t* = -0.725; z = -0.182; *p* = .470; 95% CI [-0.673, 0.309]) or sex (*t* = -1.018; z = -0.268; *p* = .311; 95% CI [-0.786, 0.248]). Participants were not found to differ significantly in producing critical lures in the same environmental context (N = 47; m = 1.57; SD = 1.26) compared to the alternative virtual context (N = 45; m = 1.44; SD = 1.14). Frequencies per sex and conditions are summarized in Table 4.

**Table 4:** Summary of central tendency measures in critical lures.

Critical lures	Condition	N	Mean	Median	SD
Men	Experimental	16	1.688	2	1.302
	Control	17	1.588	2	1.228
Women	Experimental	29	1.310	1	1.039
	Control	30	1.567	1.5	1.305

**Fig 3:**
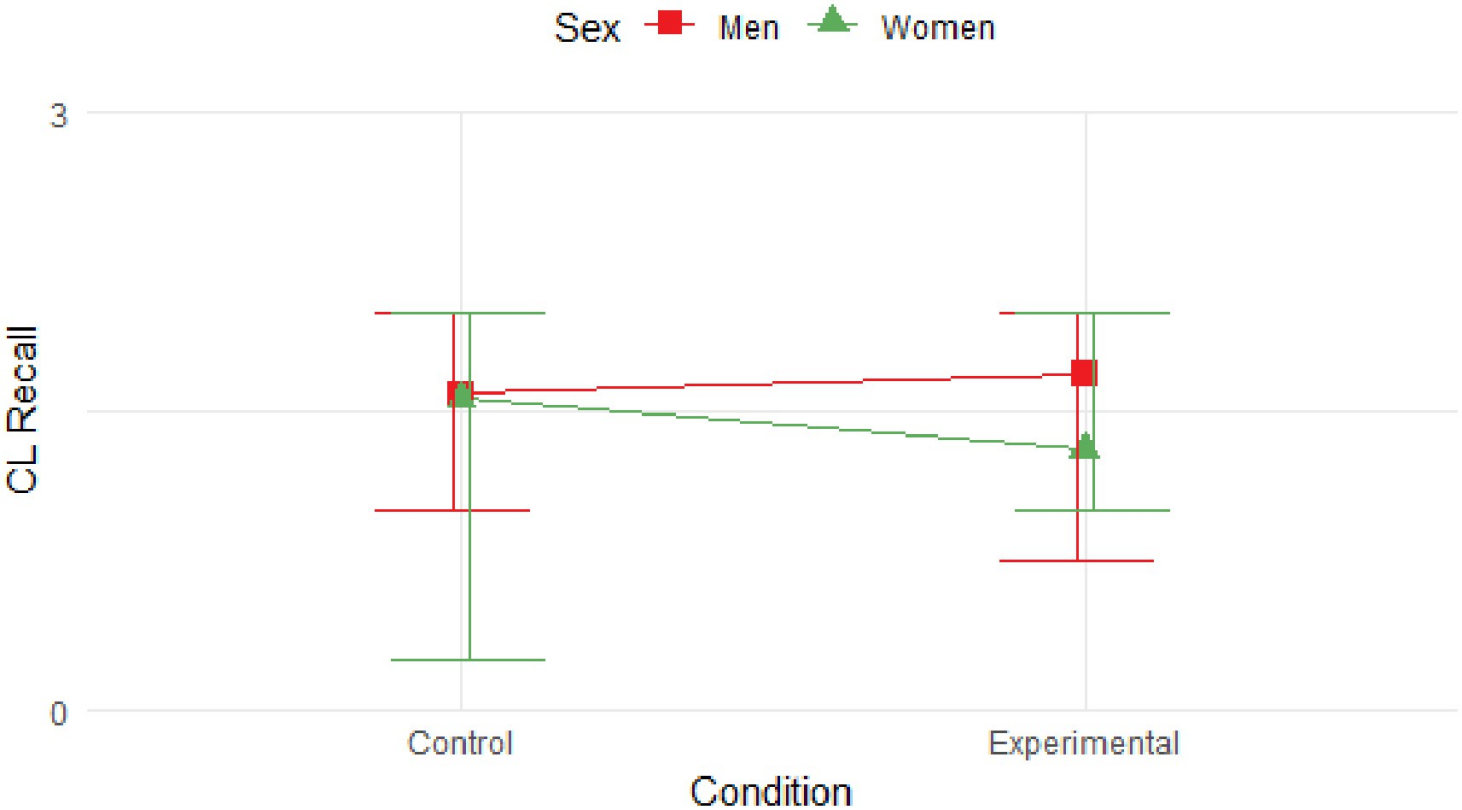
Average false recall of critical lures according to sex and condition. Men and women did not differ significantly from each other in the induction of false memories regardless of condition. False memories were also not induced by exposure to a different or the same context during word list learning and recall in general. The Y-axis expresses the sum of all falsely remembered words by the participant, while the X-axis specifies the score according to the experimental condition. Error bars represent the range of values that contain the middle 50% of the data.

Fig 3 shows that there were no significant differences between men and women in the production of critical lures in the experimental or control condition.

## 4. Discussion

The aim of this study was to explore the influence of environmental context change on memory recollection and false memory induction using state-of-the-art VR technology. Following the DRM paradigm, participants were asked to memorize 55 words while they were virtually present in an indoor virtual environment. Afterwards, they were asked to recall as many words as they could while placed in the same or a different virtual environment. Although sensory stimulation was restricted to visual stimuli, respondents were able to look around actively by moving their heads. The whole visual field was simulated, which promoted realism and the participants’ sense of agency within the experimental environment.

Contrary to expectations, memory recollection was the same regardless of the environment in which participants had learned the words (*p* = .070). Participants who were teleported into an alternative virtual environment (outdoor Environment B) did not tend to recall fewer words from the presented lists than people who had remained in the original visual scene. These results do not provide sufficient evidence for context-dependent memory recollection in virtual environments, which is in line with some previous claims in the field [23, 37] where the CDM effect has been seriously questioned. However, we identified a significant effect of biological sex on memory performance (*p* < .004), where men, on average, recalled only 16 words compared to an average of 20 words for women. This trend was observed in both the experimental as well as control conditions. For false memory induction, the gathered data did not reveal any significant effects regarding specific context or sex.

It is important to mention that this study has several limitations. The most substantial one was that the context was altered only during the recall phase. By default, both contexts should alternate during learning and testing, the so-called fully-crossed design (e.g., [38]) in order to exclude the influence, in our case, of the indoor environment on memory performance. It is possible that this study reflects a combination of effects (e.g., learning and recalling material in different contexts impairs memory performance, but if word recall was generally more effective in an outdoor environment, these two effects would cancel out). Another limitation is that we did not ask the participants, for example, about their sense of presence in VR. It seems that this aspect could significantly influence the participants’ memory performance, although it was not our primary goal to verify this relationship [22]. We suggest that further research do so. Another potential limitation to consider is the semantic association of the recalled words with the content of the presented virtual scene. Because various visually complex virtual environments may trigger different associations, it’s important to note that, for example, in this study a window could be seen in both virtual environments used, but it was visually distinct in each context, which may have provoked different levels of association. Therefore, when generating virtual experimental stimuli, it’s important to carefully consider these potential variations.

The observed word recollection differences in the different environments indicate a non-significant effect of word recall context, similar to the findings of Wälti, Woolley and Wenderoth [37]. This may be related to the size of the research sample. With this in mind, we handled the data with the use of linear modeling, where all observed trends are described and critically discussed. In any case, follow-up research on a larger sample is necessary, since present evidence on CDM in immersive virtual environments suggests existing effects of biological sex on word recall in virtual environment contexts.

The limited interaction options of participants in the virtual environments was another limitation of this study. A possible factor that may have influenced the outcome of the study is the fact that the participants in the research were only able to sit and look around the environment but were not able to move in it or interact in any other way. It has been argued that interacting with the environment generated by VR technologies could lead to improved memory performance [60]. Even though haptic interactivity with the immersive virtual environment was prevented, in accordance with the original method used in the DRM paradigm [3], we kept the interactivity (head-movement options) on an immersive and realistic, but well-controlled level. Follow up research is required that would study the specific influence of the degree of visual richness and interactivity on CDM.

Regarding the above discussed evidence, we suggest that in a virtual context, the memory abilities of men and women differ. This observation may be due to better spatial memory in men compared to women’s memory performance in autobiographical and semantic memories [42], as the task presented in this study did not involve the active use of spatial orientation to remember learning material. It suggests that this subject requires further exploration. With regard to the above-mentioned methodology, VR technology can be of great benefit in the creation of a multitude of controllable, dynamic, complex, customizable and rich virtual environments that can be presented easily with reliable measuring options.

## 5. Conclusion

This article describes original VR-based experimental research on the memory recall process. Ninety-two students of psychology wearing head-mounted VR displays were asked to memorize presented lists of words (55 words in total). Later, participants were asked to recall as many words as they could during a free recollection phase. The control group was asked to recall words while placed in the indoor virtual environment in which the words had been memorized, while the experimental group was placed in an alternative outdoor virtual environment. The number of correctly and falsely recollected words was then measured. The analysis of the data revealed there was no significant effect in the recall of words when participants were present in the virtual environment in which they had learned the words compared to word recall in an alternative virtual environment. However, there was a significant effect of sex, revealing that men recalled significantly fewer correct words than women. No effects were observed for recall of false words. A possible explanation of this finding is that learning the type of material used in this study depends on the specifics of the memory processes of biological sexes. The expected general influence of the alternative virtual environment on the higher production of correct and false memories was therefore not supported. Overall, this study is a replication of the original empirical experiment in the area of memory research extended by the use of virtual reality technologies. It uses a simple design of altering the recollection context and does not address the effect of the learning environment. In addition, it demonstrates the benefits of using VR–a suitable, cost-effective and ecologically valid tool for the research of cognitive processes, providing participants with a reality-like experience under fully controlled conditions.

## Supporting information

S1 File (ZIP)
